# Focusing on Vision Through an Environmental Lens

**DOI:** 10.1289/ehp.113-a822

**Published:** 2005-12

**Authors:** Julia R. Barrett

Our eyes are our window to the world, but for many people the view becomes dim or even darkens entirely due to visual impairment. Although the full impact of the environment on sight is unknown and significant gaps remain in our understanding of vision disorders, many reports have shown that low vision and blindness can be directly or indirectly related to environmental exposures.

Vision is described in terms of visual acuity and field of vision. Visual acuity is a measure of how well an individual sees compared with someone with normal sight—for example, a person with 20/60 vision must be within 20 feet of an object to see it as clearly as a normal-sighted person at 60 feet—and a normal field of vision is 160 to 170 degrees.

The World Health Organization (WHO) estimates that approximately 124 million people have low vision, which it defines as visual acuity between 20/60 and 20/400 with the best possible correction or a visual field of 20 degrees or less. Another 37 million people meet the WHO definition for blindness, which is visual acuity that cannot be corrected to better than 20/400 or a visual field of 10 degrees or less. An analysis published in the April 2004 *Archives of Ophthalmology* by the Eye Disease Prevalence Research Group, a consortium representing several institutions, indicates that low vision or blindness affects 3.3 million Americans over age 40 and predicts that this figure may be as high as 5.5 million by 2020. (In this study, low vision was defined as visual acuity between 20/40 and 20/200 with best correction, while blindness was defined as visual acuity of 20/200 or worse with best correction.)

According to the WHO, the rising trend is likely to be seen globally as well. An expanding population explains some of the increase, but more critically, the fastest-growing population sector comprises people older than 50. Worldwide, more than 80% of people who are blind are 50 or older, although they represent only 19% of the world’s population. Gender is also significantly associated with visual impairment. Women represent two-thirds of those with blinding eye disease, even after controlling for women’s longer life spans.

Risk also varies by race, ethnicity, and world region. Socioeconomic development often predicts regional prevalence of a disorder. Of the approximately 1.4 million children with blindness in the world, about 75% live in high-poverty areas in Asia and Africa. Among children in high- and middle-income countries, optic nerve defects, other neurological problems, and retinopathy of prematurity (a consequence of incomplete eye development) are the most common causes of blindness.

Developing nations are disproportionately affected due in large part to the burdens associated with poverty: lack of clean water and sanitation, limited or nonexistent health care, and malnutrition. Among children in low-income countries, vision problems arise mostly from complications of measles or rubella, nutritional deficiency, improper or inadequate treatment, and eye infections in the first days of life. In Tibet, an area with one of the highest prevalences of cataract, a lack of vitamin A is compounded by exposure to high-altitude ultraviolet (UV) light, soot and pathogens from indoor burning of coal and yak dung, and a dusty, windy environment. As a result, 10.9% of the total Tibetan population suffers visual impairment.

## Cataract

The primary insult to the eye is age, and one very common result of aging is cataract, in which the lens acquires color and may also become clouded or opaque. “If you live long enough, you will get cataract,” says Roger Truscott, an associate professor and senior research fellow at the Save Sight Institute at the University of Sydney in Australia. Non–age-related cataract arises from specific mutations in membrane proteins, injury, toxic or infectious exposures, and diabetes. Family studies have shown that genetics has a role in heritable cataract and may influence the development of age-related cataract, although no specific genes have been identified.

Cataract ranks as the leading cause of blindness and low vision worldwide. The WHO estimates that nearly 48% of global blindness arises from cataract. Cataract removal is one of the most common surgeries in the United States, with approximately 1 million operations performed each year. In developing countries, cataract can mean permanent blindness because sight-restoring treatment is unavailable or unaffordable for many people.

Among the suspected environmental contributors to age-related cataract are UV light exposure, cigarette smoking, and a diet low in antioxidants. Natalie Kurinij, program director of the NEI Vision Research Program, cites results published over the years from the Chesapeake Bay Waterman Study and the Salisbury Eye Evaluation Project as supportive evidence of the risk presented by UV exposure. “We’re fairly confident that sunlight exposure plays a role,” says Kurinij.

That role seems to be linked to oxidative damage, which follows the generation of free radicals. The same sort of mechanism is suspected with cataract risk associated with smoking. Smoking generates free radicals throughout the body, and those may be responsible for lens damage. The precise mechanisms by which oxidative damage to the lens occurs are still being investigated.

The relationship between free radicals and lens damage may not be direct, however. “The epidemiological data are surprisingly weak when you think that our eyes are exposed for about half of our lifetime, and it seems to make sense that a transparent tissue, designed to transmit light, should suffer ultraviolet damage,” says Truscott. The lens possesses a good UV filter system, but it decreases with age. Further, the lens’s ability to maintain low oxygen levels within its center also seems to diminish, and the lens thus becomes more susceptible to oxidative damage. Due to these changes in the lens, the compounds that serve as UV filters may bind to lens proteins, which then become more sensitive to UV radiation–induced damage.

A diet rich in free radical–scavenging antioxidants might be protective, but proof is lacking. “Vitamin data are not convincing with regard to cataract, but [vitamins] E and C could be useful. There’s still much more to know,” says Truscott. Kurinij agrees that support for nutrition’s role varies. “There have been conflicting reports from observational studies regarding the role of antioxidant nutrients and the development of cataract,” she says. “Any potential effect of antioxidant nutrients on cataract will probably depend on the nutritional status of the population to begin with.”

To illustrate the complexity of researching dietary effects on vision, Kurinij compares randomized clinical trial results from the NEI’s Age-Related Eye Disease Study to cataract studies conducted in Linxian, China. In the NEI study, high-dose antioxidant and zinc supplements over a six-year period was not associated with lens opacities in a healthy, well-nourished U.S. population. However, in the relatively nutritionally deprived Linxian subjects a multi-vitamin supplement or a supplement with riboflavin and niacin was associated with fewer cases of cataract.

Oxidative damage also figures in research conducted by Debra Schaumberg, a clinical associate scientist at Schepens Eye Research Institute and an assistant professor of medicine and ophthalmology at Harvard Medical School. Schaumberg and her colleagues reported in the April 1999 issue of the *Annals of Epidemiology* that people with higher blood levels of C-reactive protein, an indicator of systemic inflammation, had a higher incidence of cataract. “This was really the first time that anyone had shown that systemic inflammation, with no clinically detectable inflammation in the eye, increased the risk of cataract,” says Schaumberg, who notes that older people and obese persons tend to have higher levels of inflammatory activity in the body. “Obesity is one of the strongest contributors to the levels of something like C-reactive protein,” she says.

Schaumberg also identifies heavy metal exposure as a risk factor needing more research. According to a study led by Schaumberg and published in the 8 December 2004 issue of *JAMA*, low-level lead exposure appears linked to cataract formation in men. Of 642 men ranging in age from 48 to 93 years, 122 were diagnosed with cataract. Bone scans determined that the men’s long-term, low-level lead exposure was comparable to that of the general population. Men in the highest exposure group (8.17–35.0 micrograms per deciliter) had 2.5 times the risk of having cataract as the men in the lowest exposure group (1.0–3.0 micrograms per deciliter). “As far as we know, this paper . . . was really the only epidemiological study looking at heavy metals in relation to eye disease. I think it’s really an area that we don’t know much about,” she says.

At the opposite end of the age spectrum, children may have cataract at birth or develop the condition in infancy due to prenatal infection with rubella or toxoplasmosis, among other causes. Such cases need immediate treatment. One consequence of untreated cataract is nystagmus, any of a variety of involuntary movements of the eyes. Amblyopia, or “lazy eye,” is another condition found in children. “In the first weeks up until the first couple of months [of life], if vision is disturbed in both eyes that then will cause poor vision for the rest of life because nystagmus cannot be treated in any way,” says Jill Keeffe, an associate professor at the Centre for Eye Research Australia. “With cataract, it needs to be treated within weeks, whereas with amblyopia, which might develop from strabismus [drifting or crossing of one or both eyes] or from uneven refractive error between the two eyes, the window of opportunity is much longer. Obviously, the earlier, the better.”

## Retinal Disorders

Retinal disorders also pose a threat worldwide. Age-related macular degeneration (AMD) refers to damage to the area of the retina responsible for sharp central vision. It is the third most common global cause of blindness, accounting for approximately 8.7% of total blindness, and the primary cause of blindness in developed countries. In the United States, there are approximately 1.8 million people with vision loss due to AMD, and another 7.3 million are at risk. As the average age of the world’s population creeps upward, AMD will become even more significant.

As much as 30% of AMD may be related to smoking. A prospective study published in the 9 October 1996 issue of *JAMA* linked smoking with AMD, and more recently a study in the 14 April 2005 *British Journal of Ophthalmology* showed that smokers were twice as likely as nonsmokers to develop AMD. The risk declines if one stops smoking, to the point that after 20 years of not smoking former smokers have about the same level of risk as nonsmokers. Possible mechanisms of damage linked to smoking include depressed levels of antioxidants, reduced oxygen, and altered blood flow.

The effect of diet on AMD risk shares some of the same components as cataract; specifically, low-level antioxidant levels may heighten the chances of developing the disease. Obesity and high blood pressure, fat intake, and cholesterol levels also appear to increase AMD risk, but the specifics are not yet clear.

Family studies imply a genetic link, which is supported by three papers published in the 15 April 2005 issue of *Science* and a fourth published in the 2 May 2005 issue of *Proceedings of the National Academy of Sciences*. “Age-related macular degeneration is certainly a disease that’s affected by our genes,” says Timothy Stout, an associate professor of ophthalmology at the Casey Eye Institute in Portland, Oregon. “What’s interesting is that it’s also a disease that’s influenced by our environment. The link between those two has been puzzling in the past, and I think there are new studies that suggest that some of the genes that play a role in the development of macular degeneration are genes that may be involved in inflammation and our immune response.”

In early 2005, the four teams independently associated AMD with a gene coding for complement factor H, an inflammatory component. “The recent research implicating the complement factor H gene in AMD is a major breakthrough,” says Peter Humphries, a professor of medical molecular genetics at Trinity College in Dublin, Ireland. “Many studies have resulted in localizing so-called susceptibility genes to chromosomal regions, but the studies recently reported are the first to identify an actual gene. I expect that we will find out a great deal more about the so-called molecular pathology of AMD as a result of this discovery.”

Further, up to six regions within the genome have been implicated as potentially harboring AMD genes, and a second gene was reported in the November 2005 *Human Molecular Genetics*. “As yet we have very little information about this most recent gene,” says Humphries. “[However], once more is known about the mechanisms of action of such variants, we stand to know a lot more about the cause of AMD, and hence the prospects for eventual prevention will become more realistic.”

Retinal damage is also a hallmark of diabetic retinopathy, which blinds about 5 million people worldwide. The U.S. Centers for Disease Control and Prevention estimates that 13.8 million Americans have been diagnosed with diabetes and another 5.2 million have it without realizing it. Duration of diabetes and its control affect risk of diabetic retinopathy, and approximately 5.3 million Americans over age 18 have the eye condition. With diabetes rates increasing, in part due to increasing obesity, diabetic retinopathy can be expected to become more prevalent. Dietary and genetic factors may also affect its development, as may high blood pressure and high cholesterol.

“All of these things—hypertension, diabetes, hypercholesterolemia—tend to have bad effects at the level of the small blood vessels, the capillaries,” says Stout. Retinal vein blockages associated with high cholesterol, high triglycerides, and high blood pressure can create capillary-bursting pressure. The tissue then becomes hypoxic, or insufficiently oxygenated. Retinal hypoxia also occurs in diabetes when the capillary network dies through mechanisms that are not completely clear.

Hypoxia triggers production of vascular endothelial growth factor, which promotes formation of new blood vessels, but the process is disorganized. “The body tends to not build the blood vessels in the right place, so they will grow not as nicely formed capillaries in the retina, but at the optic nerve or into the center part of the eye or at the part of the eye where the drainage system is, and that causes all sorts of problems,” says Stout. Further, the poorly built new vessels leak, as may existing blood vessels. Ultimately, the retina detaches from the underlying layers, and vision is lost.

## Infections and Nutritional Deficiencies

Slightly more than 5% of global blindness arises from injury- or disease-associated corneal opacity. Distinct from this category, trachoma accounts for an additional 3.6% of global blindness. Trachoma is the most common infectious cause of vision loss and affects approximately 84 million people, primarily in remote rural areas of Africa, Asia, Central and South America, Australia, and the Middle East. This disorder arises from repeated infection by *Chlamydia trachomatis* bacteria that are spread by close contact with an infected person or by flies. After numerous infections, eyelid scarring turns the eyelashes inward, and they rake against the cornea, a condition called trichiasis. The irritation scars the cornea and eventually renders it opaque.

Infection typically starts in childhood, although the blinding effects do not occur until well into adulthood. “It’s not just one infection—it’s repeated infections,” says Keeffe. “We’ve seen scarring in the lids of preschool-aged children, . . . but it’s usually not until the forties and fifties that vision loss occurs.” In some areas, 60–90% of pre-school-age children carry active infections. Women account for 75% of late-stage blinding trachoma cases, possibly because they have greater contact with children.

In 1997 the WHO launched GET (Global Elimination of Trachoma) 2020 with the goal of eradicating trachoma altogether. A major part of GET 2020 is a primary health care plan known as the SAFE strategy. The SAFE strategy utilizes lid surgery (S), antibiotics (A) to treat active infections, facial cleanliness (F), and environmental changes (E) geared toward improving sanitation and access to clean water. A review of the SAFE strategy published in the June 2003 issue of *The Lancet Infectious Diseases* found strong support for the use of antibiotics and surgery in warding off infection and blindness, although the evidence for face washing and environmental improvements was weaker.

Onchocerciasis, also known as river blindness, is caused by *Onchocerca volvulus*, a parasite transmitted by blackflies in riverside areas. The disease is endemic in West and Central Africa, Yemen, and several South American countries. When a blackfly bites, a juvenile form of the parasite enters the body. Once mature, females release high numbers of larvae that migrate to the skin and eyes. Associated lesions form in all eye tissues except for the lens, and lead to inflammation, bleeding, secondary infections, and eventually blindness. More than 17 million people are infected with the parasite, approximately 500,000 people are visually impaired as a result, and another 250,000 are blind. Fear of infection prevents arable riverside land from being used, and local economic growth stagnates.

Global efforts at halting river blindness started with effective vector control efforts in 1974, and ongoing community-based treatment with ivermectin, an antiparasite medication, began in 1996. The disease has been reduced in most areas and may be eradicated from Latin America by 2010.

Among children, cornea-clouding vitamin A deficiency is the most common cause of preventable childhood blindness. Poor night vision is the key symptom of the very early stages of the corneal damage preceding blindness. At this stage, children can retain their vision with repeated doses of vitamin A. In late-stage vitamin A deficiency, the cornea becomes very white and cloudy, and vision loss is irreparable; cornea transplants are impossible because the tissue becomes too damaged. Immunization and good nutrition are key to preventing this form of blindness, but where these interventions are not immediately possible, doses of vitamin A make “lovely primary health interventions,” says Keeffe. In addition to supplying vitamin A supplements, health organizations also strongly promote vitamin A–rich diets. Breastfeeding provides ample vitamin A to babies, and older children and adults receive the nutrient through garden produce and supplemented foods.

## An Eye to the Future

The WHO estimates that up to 75% of all blindness is preventable. Vision 2020: The Right to Sight, a program instituted in 1999 by the WHO and the International Agency for the Prevention of Blindness, builds upon previous programs, dovetails with pre-existing efforts of many organizations, identifies remaining regional and national needs, and provides a framework for filling gaps. Through Vision 2020, a coordinated effort is under way to eliminate preventable blindness by 2020 by increasing awareness of eye disease, garnering resources for prevention and treatment, controlling major causes of avoidable blindness, training ophthalmologists and other eye care personnel to diagnose and treat the diseases specific to certain regions, and providing these specialists with necessary technology and infrastructure.

Other organizations also have carried out large-scale international programs. For example, Lions Clubs International, a service organization with a long history of combating low vision and blindness, instituted the worldwide SightFirst program with three major goals: treating and preventing diseases such as river blindness, trachoma, and cataract; providing education and training of health care workers to diagnose and treat eye disease; and constructing and equipping health care facilities. The group is currently building SightFirst II as well as collaborating with the WHO on the Project for the Elimination of Avoidable Childhood Blindness.

On a national level, the U.S. government sponsors prevention and treatment through the Department of Health and Human Services’ Healthy Vision 2010 and the NEI’s National Eye Health Education Program. Healthy Vision 2010 is part of Health People 2010, a national program to improve the health of Americans, and seeks to promote regular eye examinations for adults and children, vision screening for preschoolers, and injury prevention. There’s also a component to educate people with low vision about treatment. The National Eye Health Education Program has a more specific focus, encouraging early detection and treatment of glaucoma and diabetic eye disease, and providing education about treatment for low vision. This program provides materials that communities can use to educate the public about eye disease and the importance of early detection and treatment.

A critical gap in eliminating preventable blindness and low vision is delivery of health care. Without access to health care, opportunities are missed to diagnose problems early when treatment is most likely to be successful. Access to health care is a problem in many nations, including the United States. Stout offers diabetes as an example of the United States falling short in this regard. “That’s a huge population problem for us in the United States, because as people are falling through the health care cracks and aren’t [controlling diabetes] they’re going to get very significant blinding diabetic retinopathy at a relatively early age,” he says. “Vision loss in anybody is not a good thing, but vision loss in a relatively young, healthy person who presumably has a productive career ahead of them—it’s a real issue.”

For all that’s been learned about eye disease, there remain gaps, and new hypotheses continue to be generated. Researchers at the Schepens Eye Research Institute recently embarked on the $2.2 million, three-year Planning Grant for Research on Blinding Eye Diseases. Like other NIH “roadmap” grants, this one is designed to promote interdisciplinary collaborations regarding complex health challenges. “While experts from these areas often collaborate informally on [eye] disease, ophthalmology has remained somewhat specialized and in some ways isolated from other disciplines,” remarked Darlene Dartt, director of scientific affairs at Schepens Eye Research Institute, in announcing the grant. “This is really the first federal program to formalize collaboration.” In learning more about the cascade of events occurring in other parts of the body in diseases ranging from Alzheimer disease to rheumatoid arthritis, researchers might gain some insight into the processes of blinding eye diseases.

## Figures and Tables

**Figure:**
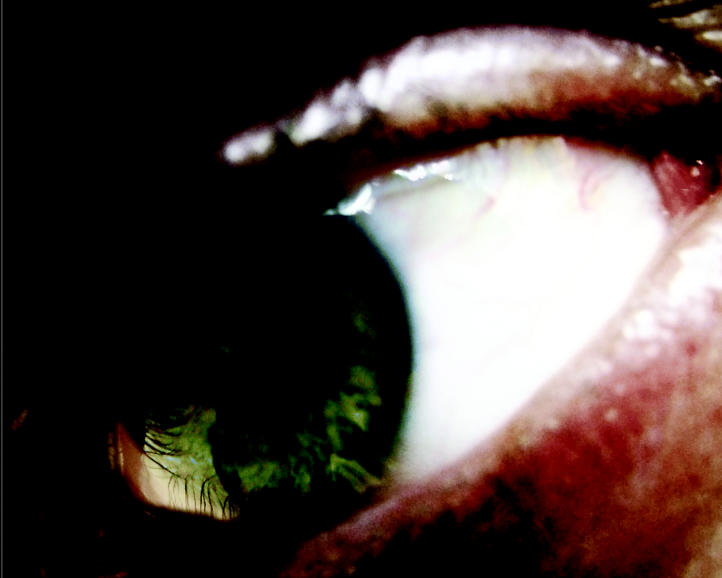


**Figure:**
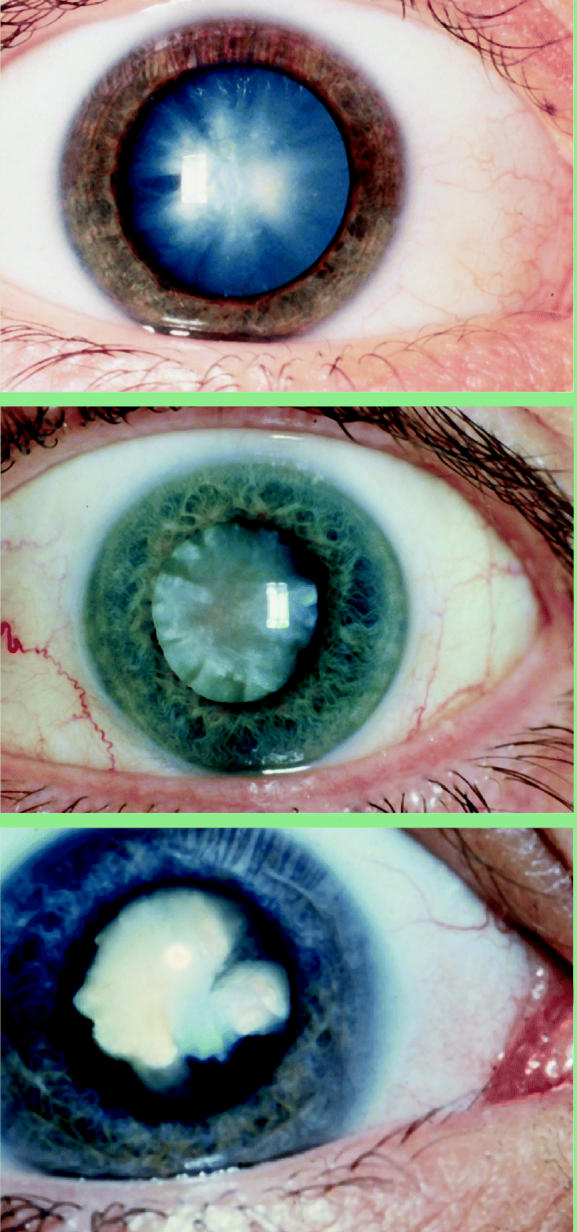
Clouding our vision. Cataract, the leading cause of blindness worldwide, arises from a combination of factors including genetics, age, and environmental exposures. Though largely treatable, poor access to health care leaves many around the world to suffer. (Top to bottom) An acute sudden-onset cortical cataract in a person with type 1 diabetes; a hypermature age-related cataract; a white congenital cataract.

**Figure:**
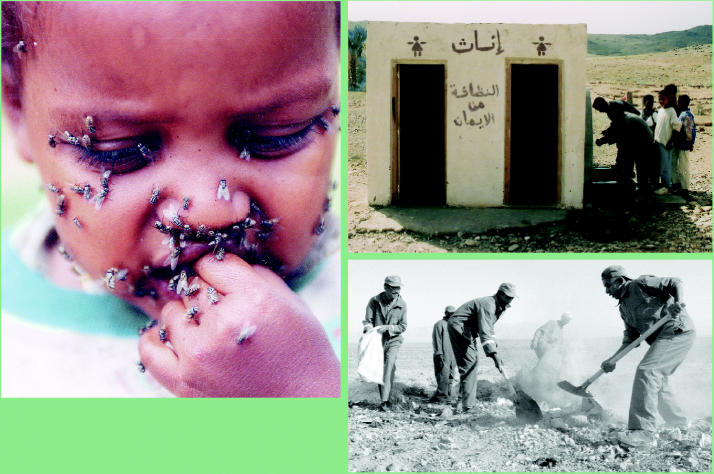
Insight into the problem. Flies attracted to eye secretions are one way trachoma is transmitted (above). In its bid to eradicate this disease by 2020, the WHO encourages facial cleanliness (right top) and improvements in sanitation such as burying waste (right bottom).

**Figure:**
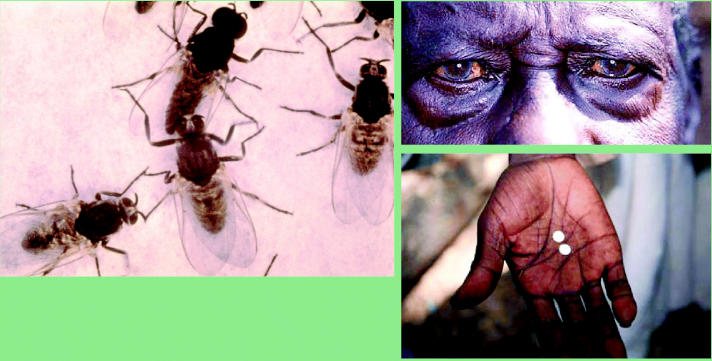
Focusing on onchocerciasis. When parasites spread by blackflies (above) enter the body, their larvae migrate to the eyes and skin, causing lesions that result in inflammation, bleeding, and severe visual impairment or blindness if left untreated treated (right top). Treatment with ivermectin (right bottom) has reduced the disease in most areas and nearly eradicated it in some.

**Figure:**
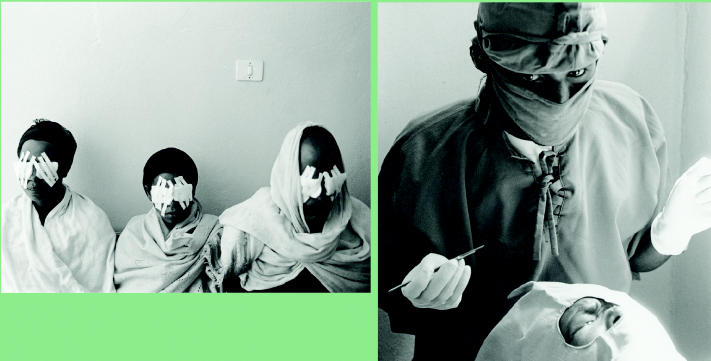
Seeing a way past trachoma. A 15-minute procedure performed by a doctor or trained nurse using local anesthetic can reverse the conditions that cause trachoma and reduce the risk of blindness. Globally, however, lack of access to basic health care keeps many patients in the dark.

